# Synthesis and antitumor activity of dolutegravir derivatives bearing 1,2,3-triazole moieties

**DOI:** 10.1186/s13065-024-01205-3

**Published:** 2024-05-07

**Authors:** Xixi Hou, Longfei Mao, Yajie Guo, Lan Wang, Lizeng Peng, Huili Wang, Jianxue Yang, Sanqiang Li, Yue-Ming Li

**Affiliations:** 1 Department of Pharmacy, The First Affiliated Hospital, and College of Clinical Medicine of Henan University of Science and Technology, Luoyang, 471003 China; 2 College of Basic Medicine and Forensic Medicine, Henan University of Science and Technology, 263 Kaiyuan Road, Luoyang, 471003 China; 3 State Key Laboratory of Medicinal Chemical Biology, College of Pharmacy and Tianjin Key Laboratory of Molecular Drug Research, Nankai University, Haihe Education Park, 38 Tongyan Road, Tianjin, 300350 China; 4 Department of Emergency, The Eighth Affiliated Hospital, Sun Yat-Sen University, Shenzhen, 518033 China; 5 Institute of Agro-Food Science and Technology, Key Laboratory of Agro-Products Processing Technology of Shandong Province, Key Laboratory of Novel Food Resources Processing Ministry of Agriculture, Shandong Academy of Agricultural Sciences, Jinan, 250100 China; 6 University of North Carolina Hospitals, 101 Manning Dr, Chapel Hill, Orange County, NC 27599 USA

**Keywords:** Dolutegravir, 1, 2, 3-triazole, Synthesis, Antitumor, Autophagy

## Abstract

Modification of marketed drugs is an important way to develop drugs because its safety and clinical applicability. Oxygen-nitrogen heterocycles are a class of important active substances discovered in the process of new drug development. Dolutegravir, an HIV drug with a nitrogen-oxygen heterocycle structure, has the potential ability to inhibit cell survival. In order to find and explore novel anti-tumor drugs, new dolutegravir derivatives bearing different 1,2,3-triazole moieties were prepared via click reactions. In vitro biological experiments performed in several lung cancer cell lines suggested that these novel compounds displayed potent anti-tumor ability. Especially, the compound 9e with a substituent of 2-methyl-3-nitrophenyl and the compound 9p with a substituent of 3-trifluoromethylphenyl were effective against PC-9 cell line with IC_50_ values of 3.83 and 3.17 µM, respectively. Moreover, compounds 9e and 9p were effective against H460 and A549 cells. Further studies suggested that compounds 9e and 9p could induce cancer cell apoptosis in PC-9 and H460, inhibit cancer cell proliferation, change the cell cycle, and increase the level of reactive oxygen species (ROS) which further induce tumor cell apoptosis. In addition, compounds 9e and 9p increased LC3 protein expression which was the key regulator in autophagy signaling pathway in PC-9 cells. Compound 9e also showed low toxicity against normal cells, and could be regarded as an interesting lead compound for further structure optimization.

## Introduction

Lung cancer is the leading cause of cancer-related deaths globally, posing a serious threat to human health [1] Drug therapy is a crucial approach in the treatment of lung cancer, but traditional drugs often result in severe side effects and drug resistance. Therefore, the development of new lung cancer drugs aimed at reducing treatment side effects and resistance is essential. This effort aims to enhance treatment tolerability, ultimately improving the quality of life for patients. Such advancements carry significant societal significance and economic value [2]. Over the years, endeavors have focused on discovering drugs that either provide superior alternatives to existing standards or introduce entirely novel mechanisms of action. Despite the progress in antitumor drug development, an ongoing imperative exists to uncover and design agents that are not only safer but also more effective, minimizing side effects and mitigating drug resistance. The modification of marketed drugs is an important way to develop drugs because this approach ensures both safety and clinical applicability.

Human immunodeficiency virus (HIV) is a lentivirus that infects cells of the human immune system. By damaging the body’s immune system, the virus causes a variety of diseases including cancer, and ultimately threatens the lives of patients [3, 4]. After a patient is infected with HIV, the virus cannot be completely eliminated from the body, and one can only rely on drug treatments to reduce the viral load in body. With the effective treatment of antiretroviral virus, AIDS-related opportunistic infections have been effectively controlled. However, with the prolongation of disease courses, malignant tumors have become the main cause of death in affected individuals [5, 6]. In patients with HIV infections, cancers exhibit accelerated growth and heightened aggressiveness. Studies showed that, comparing with the general population, people infected with HIV are 500 times more likely to be diagnosed with Kaposi sarcoma, 12 times more likely to be diagnosed with non-Hodgkins lymphoma, and, among women, 3 times more likely to be diagnosed with cervical cancer [7, 8]. It would be of high interest to develop new agents which could effectively suppress the HIV infection or HIV replication on one hand, and could significantly inhibit the growth of tumor cells on the other [9].

HIV integrase inhibitors are an important class of anti-AIDS drugs, and can effectively inhibit the replication process of retrovirus and block the integration of virus DNA into the host chromosome DNA [10]. It would be possible to develop new agents exhibiting both the antiviral and antitumor activities by introduction of new functional groups into such anti-HIV agents. One can also take the advantages such as safety or the pharmacokinetic issues of a clinically used drug when using known drug as a starting point [11, 12].

**Fig. 1:**
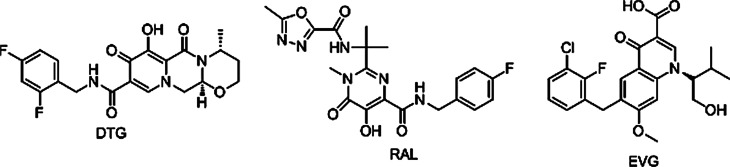
The structures of DTG, RAL and EVG

Dolutegravir (DTG), as a class of HIV integrase inhibitors approved by FDA priority [13], has shown strong antiviral and anti drug-resistant properties [14]. In the treatment of patients with first-time HIV infection, DTG taken once a day is comparable to that of Raltegavir (RAL) taken twice a day. The results of preclinical study shows that DTG has little toxicity, and no obvious fertility toxicity or teratogenic toxicity was found when the dose of DTG was 27 times greater than the clinical dose [15]. The results of clinical studies shows that DTG is better than control drug in the treatment of HIV first-infected people and has better effect on the patients who failed treatment without use of integrase inhibitors. Good responses were also found in adult patients who were resistant to RAL or EVG [16] (Fig. 1).

Oxygen-nitrogen heterocycles are a class of important active substances discovered in the process of new drug development. And DTG is an drug with a nitrogen-oxygen heterocycle structure. The outstanding performance of DTG in the treatment of HIV infections made it an ideal starting point for the development of new chemical entities which would be expected to show both antiviral and antitumor activity. In our previous report we showed that introducing 1,2,3-triazole moiety to dolutegravir produced a new dolutegravir derivative which could inhibit proliferation and induce apoptosis of non-small cell lung cancer cells via calcium signaling pathway [17]. Encouraged by this preliminary result, we wish to further study the possibility of using dolutegravir derivatives as new antitumor agents.

As an important class of nitrogen-containing heterocyclic compounds, 1,2,3-triazoles can be easily, efficiently and quickly prepared by click reaction [18]. The unique features of such hetereocycles made them ideal functional groups for the modification of a known drug, or as structural surrogates for other functional groups in a known drug [19–22]. Previous studies have found effective anti-tumor abilities by combining 1,2,3-triazoles with marketed drugs include icotinib, zidovudine, and pomadomide [23, 24]. Based on our previous research focusing on the synthesis of 1,2,3-triazole analogues with known drugs, which exhibited promising anticancer activity, we sought to explore the antitumor activity of dolutegravir and 1,2,3-traizoles combination. In this regard, a series of 1,2,3-triazole derivatives were designed and prepared using dolutegravir as the parent nucleus according to the principle of bioactive substructure splicing. Subsequently, comprehensive evaluations of their biological activity especially the antitumor activity against lung cancer cell lines were measured. The aim of our study is to discover more efficient anticancer agents for treating lung cancer.

## Results and discussion

**Scheme 1:**
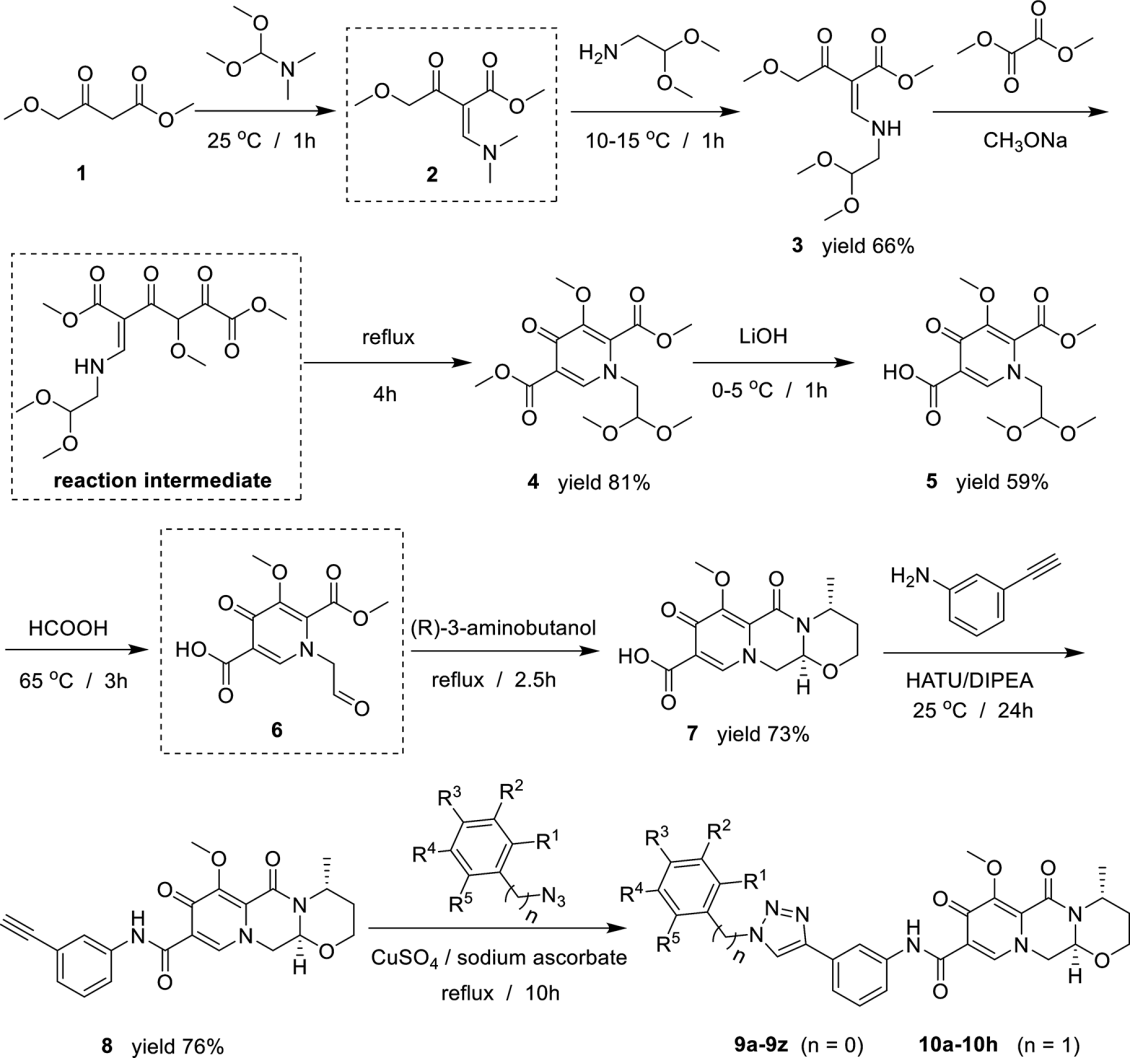
Preparation of compounds 9 and 10

The preparation of the target products started from commercially available methyl 4-methoxyacetoacetate (1). Condensation of 1 with DMF-DME gave compound 2 which reacted with aminoacetaldehyde dimethyl acetal to give compound 3. Compound 3 reacts with dimethyl oxalate to produce an reaction intermediate under room temperature conditions, and then undergoes cyclization under heating conditions to yield compound 4. Partial hydrolysis of 4 with lithium hydroxide gave carboxylic acid 5 which was further hydrolyzed with HCOOH to give 6. Reaction of 6 with (*R*)-3 aminobutanol gave compound 7 which was converted to compound 8 using HATU/DIPEA as the coupling agent. Click reaction of 8 with azide compounds bearing different substituents furnished the synthesis of target compounds 9 and 10 (Scheme 1 and Table 1). The structures of the target compounds were confirmed with ^1^H and ^13^C nuclear magnetic resonance spectroscopy.

**Table 1:** R-group of compounds 9 and 10

Compd no.	*n*	*R* ^1^	*R* ^2^	*R* ^3^	*R* ^4^	*R* ^5^
9a	0	F	H	H	H	H
9b	0	OCH_3_	H	NO_2_	H	H
9c	0	CF_3_	H	H	H	H
9d	0	H	H	CF_3_	H	H
9e	0	CH_3_	NO_2_	H	H	H
9f	0	H	CH_3_	H	H	H
9 g	0	H	H	F	H	H
9 h	0	H	H	OCH_2_CH_3_	H	H
9i	0	OCF_3_	H	H	H	H
9j	0	H	OCH_3_	OCH_3_	H	H
9k	0	CH_2_CH_3_	H	H	H	H
9 L	0	H	OCH_3_	H	H	H
9 m	0	H	H	CH_2_CH_3_	H	H
9n	0	H	H	C(CH_3_)_3_	H	H
9o	0	H	H	H	H	H
9p	0	H	CF_3_	H	H	H
9q	0	CF_3_	H	H	CF_3_	H
9r	0	H	CF_3_	H	CF_3_	H
9s	0	CH_3_	H	H	H	H
9t	0	OCH_3_	H	H	H	H
9u	0	Br	H	H	H	H
9v	0	Cl	H	H	H	H
9w	0	I	H	H	H	H
9s	0	H	H	OCH_3_	H	H
9y	0	H	H	CH_3_	H	H
9z	0	CH_3_	H	CH_3_	H	CH_3_
10a	1	H	Br	H	Br	H
10b	1	H	H	H	H	H
10c	1	Br	H	H	H	H
10d	1	H	OCH_3_	H	H	H
10e	1	H	Br	H	H	H
10f	1	F	H	H	H	H
10 g	1	CH_3_	H	H	H	H
10 h	1	CF_3_	H	H	H	H

### Compounds 9 and 10 suppressed cancer cell viability

At first, CCK8 assay was carried out to evaluate the anti-proliferative activity of dolutegravir-1,2,3-triazole derivatives against different cancer cell lines. The results of cell viability of three lung cancer cell lines A549, PC-9 and H460 in the presence of compounds 9 and 10 are summarized in Table 2. Cells were treated with different compounds at the concentration of 20 µM for 48 h. Preliminary results suggested that most of the compounds exhibited strong effects on lung cancer cell lines H460 and PC-9 and weak effects on A549.

**Table 2:** Inhibition performance of selected tumor cell lines by the compounds 9 and 10

Compd no.	Cell viability (100%), 20 µM, 48 h		
	A549	PC9	H460
9a	95.90	52.03	47.20
9b	92.60	104.86	98.64
9c	9.48	13.91	32.97
9d	82.37	105.15	69.71
9e	9.14	24.05	30.94
9f	33.47	8.80	39.20
9 g	91.65	88.64	97.85
9 h	77.55	71.96	52.87
9i	17.41	18.95	8.52
9j	87.42	85.93	92.89
9k	83.48	23.40	18.72
9 L	73.36	47.77	20.32
9 m	65.08	63.65	62.63
9n	67.60	59.44	24.58
9o	78.59	94.56	87.72
9p	10.85	2.11	5.63
9q	54.85	18.17	17.63
9r	90.73	98.55	117.07
9s	82.07	65.73	48.37
9t	7.32	4.56	3.27
9u	64.09	43.88	31.00
9v	54.06	47.42	38.13
9w	57.94	55.21	30.63
9x	69.21	78.11	108.05
9y	85.43	79.67	94.78
9z	91.32	73.07	55.57
10a	87.40	103.57	82.32
10b	87.18	96.85	55.00
10c	68.05	50.54	41.79
10d	89.00	83.17	77.21
10e	96.13	49.85	28.88
10f	82.22	61.10	32.43
10 g	71.58	54.61	35.98
10 h	77.72	66.77	28.10
DTG	108.82	91.66	27.24

Different cells were treated with 20 µM compounds and DTG. After 48 h treatment, cell viability was measured using a CCK8 assay kit. Data are presented as mean ± SE

*P < 0.05

Based on these preliminary results, compounds 9c, 9e, 9f, 9i, 9k, 9 L, 9p, 9q, 9t, 9u, 9v, 9w, 10c, 10e which were effective to most of the cell lines were chosen for the measurement of the half-maximal inhibitory concentration (IC_50_). The results are given in Table 3. As shown in Table 3, compounds 9e and 9p gave promising inhibitory activities against A549, PC-9 and H460 cell lines with IC_50_ values of 8.72 ± 0.11 and 12.97 ± 0.32, 3.83 ± 0.73 and 3.17 ± 0.18, 11.76 ± 2.31 and 10.69 ± 0.48µM, respectively. HRM (Human Renal Mesangial) cell line was used as a control to study the toxicity of these compounds against normal human cells. We discovered compounds that exhibit inhibitory effects on lung cancer cells, with good safety profiles in HRM cells; the half-maximal inhibitory concentration is generally greater than 50 µM.

**Table 3:** The half-maximal inhibitory concentration (IC_50_) of the chosen compounds

Compd no.	IC_50_ (µM), 48 h			
	A549	PC9	H460	HRM
9c	9.06 ± 0.88	4.03 ± 0.59	11.42 ± 0.59	> 50
9e	8.72 ± 0.11	3.83 ± 0.73	11.76 ± 2.31	> 50
9f	27.75 ± 0.56	4.67 ± 0.26	19.58 ± 0.32	> 50
9i	11.06 ± 1.07	4.53 ± 0.31	8.24 ± 2.06	> 50
9k	20.67 ± 0.23	9.87 ± 0.19	12.93 ± 0.80	> 50
9 L	44.34 ± 1.21	9.20 ± 0.65	25.48 ± 0.43	43.29 ± 3.27
9p	12.97 ± 0.32	3.17 ± 0.18	10.69 ± 0.48	35.55 ± 10.77
9q	13.63 ± 1.79	6.78 ± 0.31	16.50 ± 0.57	> 50
9t	17.34 ± 0.73	6.75 ± 1.43	14.16 ± 0.16	> 50
9u	> 50	7.77 ± 0.37	37.32 ± 2.25	> 50
9v	> 50	11.55 ± 0.48	16.19 ± 1.29	> 50
9w	33.66 ± 0.68	9.94 ± 0.36	14.71 ± 2.83	> 50
10c	> 50	12.05 ± 0.46	15.37 ± 1.38	> 50
10e	> 50	10.12 ± 0.71	9.44 ± 1.53	> 50

Cells were treated with different concentrations of compounds (0, 0.5 µM, 2 µM, 8 µM, 16 µM, 32 µM). After 48 h treatment, cell viability was measured using the CCK8 assay kit to calculate the inhibition percentage. Then the IC50 value was calculated using the GraphPad Prism software. Data are presented as mean ± SE

*P < 0.05

### Inhibition of cancer cell proliferation by compounds 9e and 9p

**Fig. 2:**
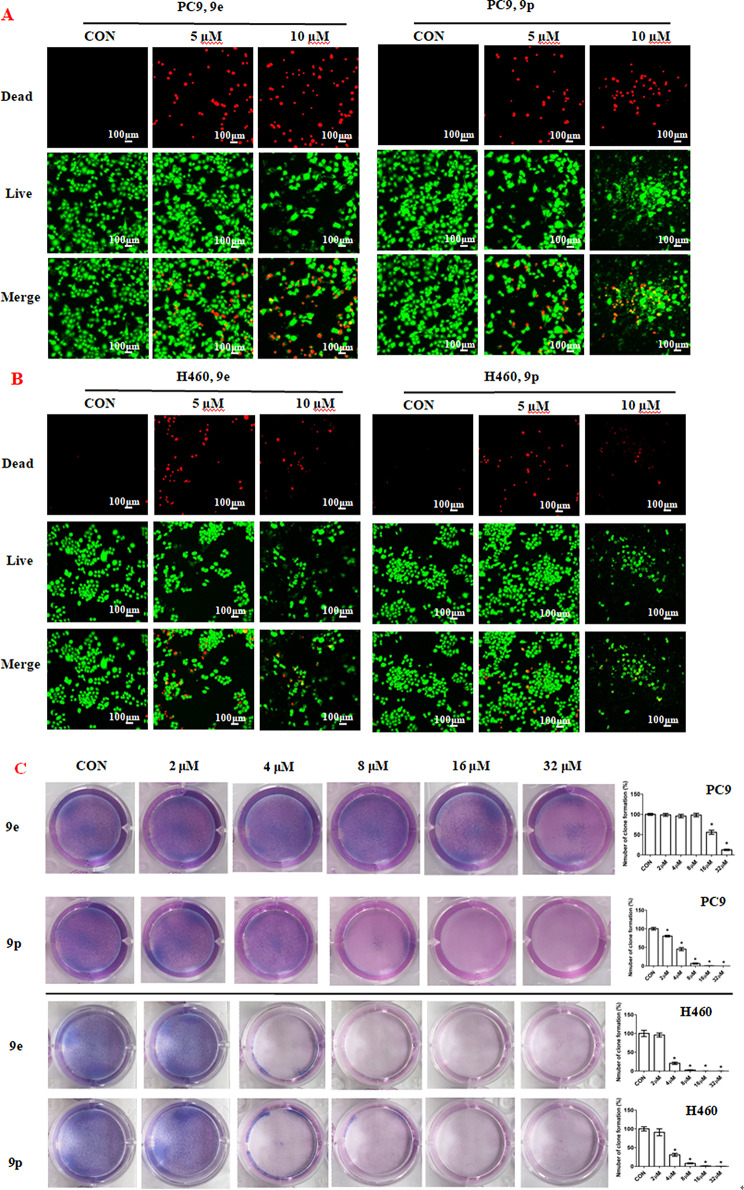
Inhibition of proliferation of cancer cells by 9e and 9p. **(A)** Fluorescence images stained with the LIVE/DEAD kit of PC-9 cells treated with 5 µM, 10 µM and 20µM of 9e and 9p. **(B)** Fluorescence images stained with the LIVE/DEAD kit of H460 cells treated with 5 µM, 10 µM and 20 µM of 9e and 9p. **(C)** Plate clone staining of PC-9 cells and H460 cells being treated with different concentrations of 9e and 9p. Data are presented as mean ± SE. *P < 0.05

LIVE/DEAD staining experiments were carried out to evaluate the anti-proliferative activity of dolutegravir derivatives 9e and 9p against PC-9 and H460 cell lines. The tumor cells were treated with 5 µM, 10 µM or 20 µM of 9e and 9p for 24 h. The live and dead cells were photographed and counted. The results are given in Fig. 2. As shown in Fig. 2, the number of live cells of PC-9 decreased significantly in a dose dependent manner after being treated with 9e. The ratio of dead/live cells was also increased significantly with the increase of concentration (Fig. 2A). In the case of 9p, the number of live cells of PC-9 also decreased with the increase of concentration (Fig. 2A). The ratio of dead/live cells was increased compared with untreated group but not completely in a dose dependent manner (Fig. 2A). The possible reason was that at the concentration of 20 µM, cell proliferation was largely suppressed, resulting in few living cells remaining. Similar results were observed in H460 cells after being treated with 9e or 9p (Fig. 2B).

The plate clone formation experiments were also carried outto further confirm the effects of dolutegravir derivatives on cell proliferation. Compounds 9e and 9p with different concentrations (0, 2, 4, 8, 16 and 32 µM) were added to the cells. Preliminary results suggested that 9e and 9p showed anti-proliferative activity against cell lines PC9 and H460 in a dose-dependent manner (Fig. 2C).

### Influences of 9e and 9p on the apoptosis of cancer cells

**Fig. 3:**
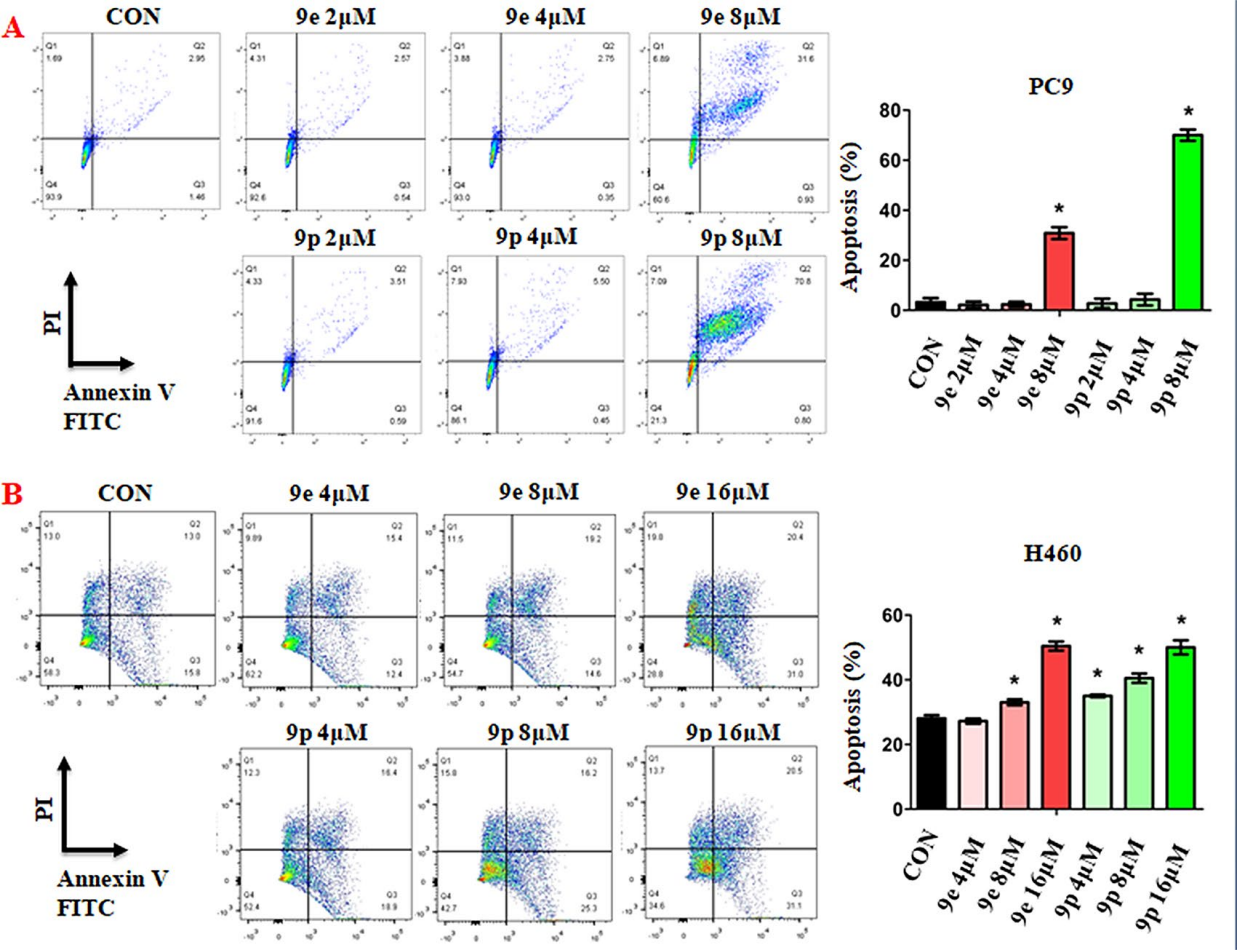
Apoptosis of cancer cells induced by 9e and 9p. (A) Apoptotic cells of PC-9 cells treated with 9e and 9p determined by flow cytometry. (B) Apoptotic cells of H460 cells treated with 9e and 9p determined by flow cytometry. Data are presented as mean ± SE. *P < 0.05

Further, the effects of 9e and 9p on the apoptosis of tumor cells were studied to get details of the suppression of cell proliferation caused by these compounds. PC-9 and H460 cells treated with different concentrations of 9e or 9p were stained with Annexin V-FITC and PI, and the numbers of apoptosis cells were analyzed by the flow cytometry. The results are given in Fig. 3. As illustrated in Fig. 3, for PC-9 cells, both 9e and 9p exhibited significant effects on cell apoptosis at the concentration of 8 µM, while little influence at the concentrations of 2 µM or 4 µM (Fig. 3A). H460 cells that treated with 8 µM and 16 µM of 9e showed significant increased apoptosis (Fig. 3B). Moreover, compound 9p at concentrations of 4 µM, 8 µM and 16 µMsignificantly increased apoptosis of H460 cells (Fig. 3B).

### Influences of 9e and 9p on the cell cycles of cancer cells

**Fig. 4:**
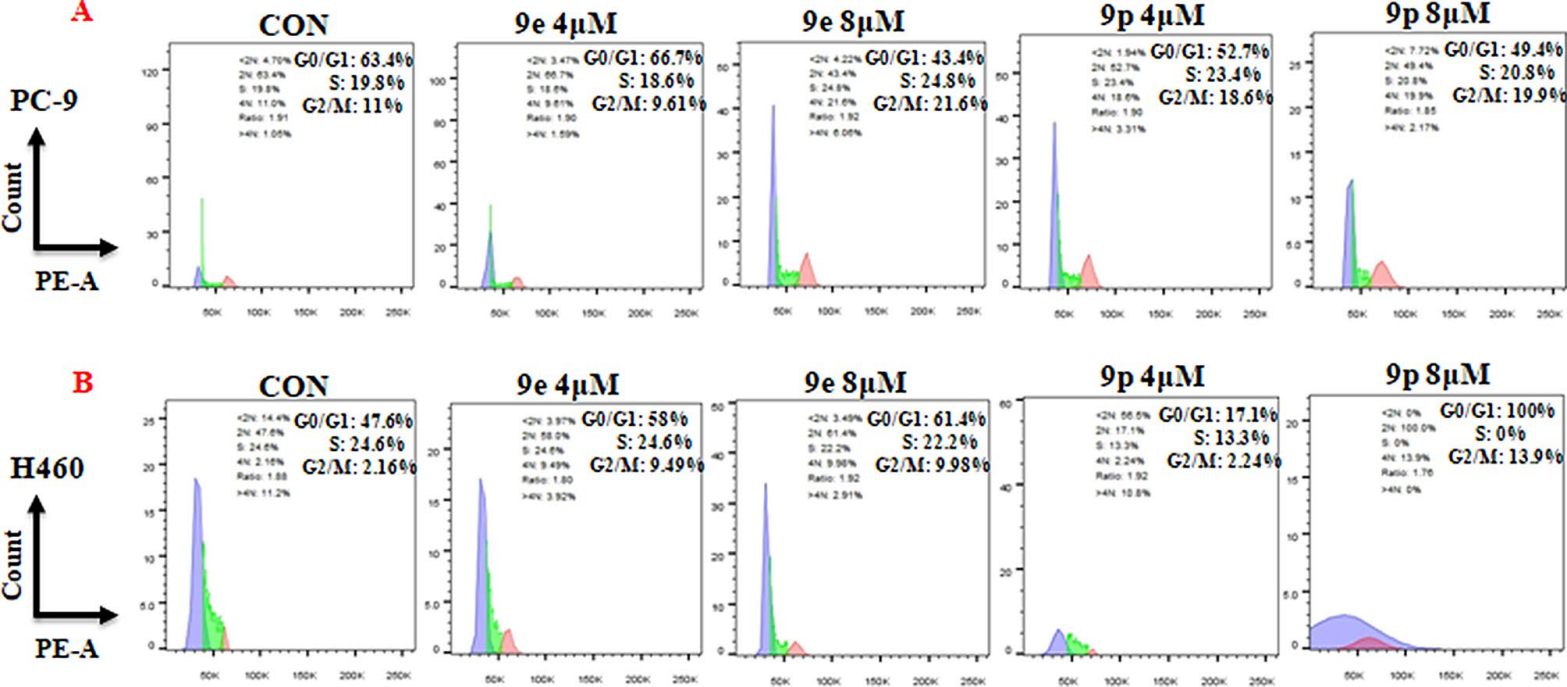
Influences of 9e and 9pon cell cycles of tumor cells. **(A)** Flow cytometry analysis of PC-9 cells treated with 9e and 9p compound for 48 h. **(B)** Flow cytometry analysis of H460 cells treated with 9e and 9p for 48 h. Data are presented as mean ± SE. *P < 0.05

Cancer cell lines PC-9 and H460 were then treated for 48 h with 9e or 9p at different concentrations to investigate the effects of these compounds on regulating the cell cycle. Cell cycle distributions were analyzed using the flow cytometry technique. The results are given in Fig. 4. As shown, for PC-9 cells, 4 µM of 9e treatment had moderate effects on cell cycle while 8 µM of 9e decreased G0/G1 phase and increased S and G2/M phase (Fig. 4A). Compound 9p at the concentrations of 4 µM and 8 µM all induced an S and G2/M cell cycle arrest, as demonstrated by the decreased G0/G1 and increased S and G2/M phase (Fig. 4A). For H460 cells, 9e treatment increased G2/M phase and had little influences on G0/G1 or S phase (Fig. 4B). Compound 9p could significantly decrease the S phase compared with untreated cells (Fig. 4B).

### Compounds 9e and 9p triggered reactive oxygen species generation in tumor cell lines

**Fig. 5:**
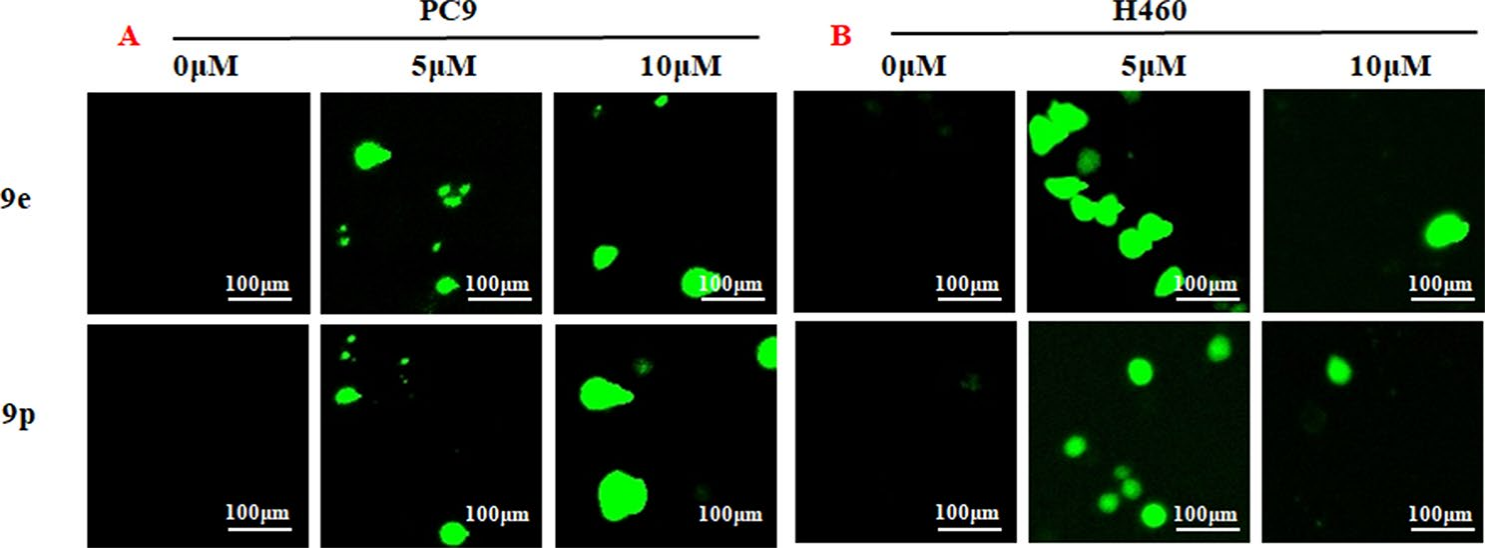
Compounds 9e and 9p triggered reactive oxygen species generation in tumor cell lines triggered by 9e and 9p. **(A)** ROS detection staining by DCFH-DA in PC-9 cells treated with 9e and 9p at the concentrations of 5 µM and 10 µM. **(B)** ROS detection staining by DCFH-DA in H460 cells treated with 9e and 9p at the concentrations of 5 µM and 10 µM

Reactive oxygen species could induce cell death and suppress cell growth and proliferation [25, 26]. To assess the role of compounds 9e and 9p in ROS generation, different cancer cell lines were treated with 9e or 9p for 24 h. DCFDA was used to stain cells and ROS was detected with fluorescent microscope. The results are given in Fig. 5. As observed in Fig. 5, ROS generation was significantly increased when PC-9 and H460 cell lines were treated with 9e or 9p.

### Autophagy signalling pathway was stimulated by 9e and 9p

**Fig. 6:**
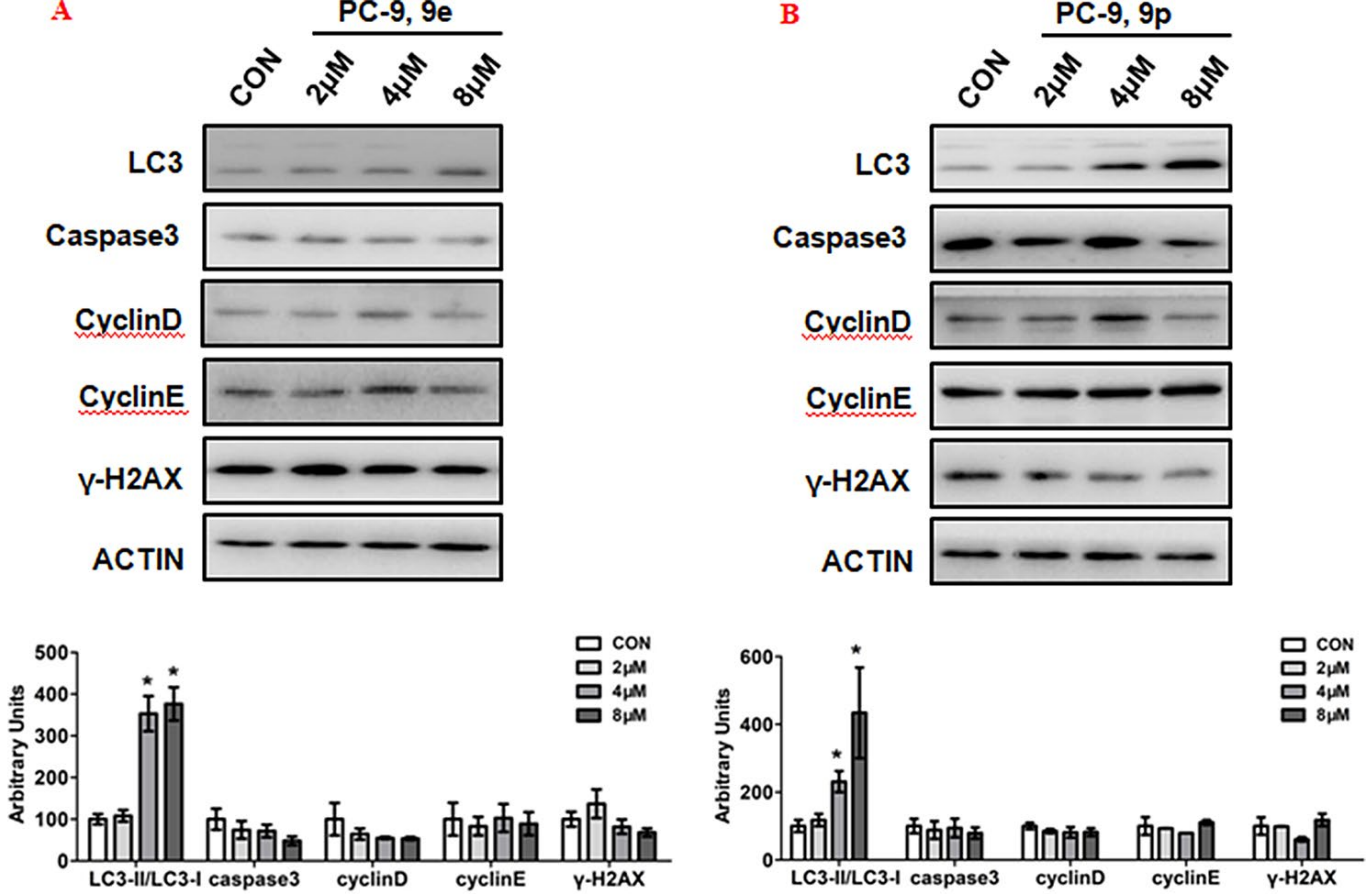
Influences of 9e and 9p on the expression of key signalling pathway proteins. (A) Western blotting of LC3, caspase3, Cyclin D, Cyclin E, γ-H2AX in PC-9 cells treated with 9e. (B) Western blotting of caspase3, Cyclin D, Cyclin E, γ-H2AX in PC-9 cells treated with 9p. Top, western blot; bottom, quantitative measurements relative to ACTIN. Data are presented as mean ± SE. *P < 0.05. The blots were cropped from different parts of the same gel or different gels, and the blots were processed in parallel. The full length original blot was included in the supplementary information

To determine the role of compounds 9e and 9p on regulating the development of cell proliferation, expressions of key proteins which were involved in cell growth progresses such as autophagy, apoptosis, cell cycle and DNA damage were examined. The results are summarized in Fig. 6. Ubiquitin like molecule light chain 3 (LC3) is a key marker of autophagy. Our preliminary study suggested that the expression of LC3 was significantly increased when PC-9 was treated with 9e or 9p. Caspase3, which is one of the key proteins in regulating apoptosis, however, was not changed in tumor cells after treated with 9e or 9p. Cell cycle regulation proteins such as CyclinD, CyclinE or β-catenin were also not affected when PC-9 was treated with 9e or 9p. Further, H2AX variant histone (γ-H2AX), which was responsible for DNA damage, was also not affected when PC-9 were treated with 9e or 9p.

## Conclusions

In summary, we have shown that conventional click reactions between dolutegravir and different azides provided derivatives bearing different 1,2,3-triazole moieties. These compounds exhibited interesting anti-tumor activities. Especially, compounds 9e and 9p were active against PC9 with IC_50_ values of 3.83 and 3.17 µM, respectively. Further study showed that these two compounds could induce apoptosis in PC-9 and H460 cells, inhibit tumor cell clone formation, and increase the level of reactive oxygen species (ROS) to further stimulate tumor cell apoptosis. These two compounds could also activate the LC3 signaling pathway to induce autophagy in PC-9 cells. Compound 9e also showed low toxicity against normal cells. The easy availability, the promising bioactivity against tumor cells and the relatively low toxicity of the compounds against normal cells made them ideal leads for further structure optimization.

## Experimental

### Materials and chemistry

All the reagents and solvents were obtained from commercially available sources. The ^1^H NMR and ^13^C NMR spectra were acquired in a DMSO-d_6_ solution using a Bruker 400 MHz or 600 MHz NMR spectrometer.

Dolutegravir-1,2,3-triazole compounds was synthesized in-house. Dimethylsulfoxide (DMSO) was obtained from Sigma-Aldrich (St. Louis, Missouri, USA). Dulbecco’s modified Eagle medium (DMEM), RPMI 1640 Medium, Fetal bovine serum (FBS) and penicillin/streptomycin were purchased from Gibco (Grand Island, NY, USA). Enhanced Cell Counting Kit-8, Calcein/PI Live/Dead Viability Assay Kit, Giemsa dye and Reactive Oxygen Species (ROS) Assay Kit were obtained from Beyotime Biotechnology (Shanghai, China). Annexin V-FITC/Propidium iodide (PI) staining kit and Matrigel Matrix were provided by BD Biosciences (Franklin Lake, New Jersey, USA).

Human lung cancer cell lines A549, PC-9 and H460 were all obtained from ATCC. Cells were cultured in DMEM or RPMI 1640 medium containing 10% FBS and 1% penicillin/streptomycin at 37 °C with a 5% CO_2_-humidified atmosphere.

### Preparation of compounds 9 and 10

#### Synthesis of methyl (Z)-2-(((2,2-dimethoxyethyl)amino)methylene)-4-methoxy-3-oxobutanoate (3)

To methyl 4-methoxyacetoacetate (compound 1, 150 g,1.0 mol) was added dropwise DMF-DMA (120 g, 1.0 mol) at room temperature in a period of 20 min. The color of the solution became black. The reaction temperature was maintained at 25 °C, and the reaction mixture was stirred for 1 h. The course of the reaction was monitored with TLC. After the completion of the reaction, the reaction mixture was cooled to 10 to 15 °C. Aminoacetaldehyde dimethylacetal (115 g, 1.1 mol) was added slowly dropwise in a period of 30 min. The reaction temperature was maintained below 30 °C with water bath. After the completion of addition, the reaction mixture was maintained at 10–15 °C for another 30 min with the formation of large amount of solid. Methyl tert-butyl ether (100mL) was added, and the suspension was stirred slowly for 15 min. The solid was collected via filtration, was washed twice with methyl t-butyl ether (50mL each), and was dried to give compound 3 as a solid (174 g, 66%). ^1^H NMR (400 MHz, DMSO-d_6_): δ 10.95 (s, 1H), 7.95 (d, *J* = 16.0 Hz, 1H), 4.56 (s, 2H), 4.40 (t, *J*^1^ = 4.0 Hz, *J*^2^ = 4.0 Hz, 1H), 3.70 (s, 3 H), 3.45 (s, 3 H), 3.43 (s, 2H), 3.41 (s, 3 H). ^13^C NMR (100 MHz, CDCl_3_): δ 196.89, 166.93, 160.94, 102.73, 98.37, 59.14, 54.91, 51.88, 50.87.

#### Synthesis of dimethyl 1-(2,2-dimethoxyethyl)-3-methoxy-4-oxo-1,4-dihydropyridine-2,5-dicarboxylate (4)

To a solution of compound **3** (26 g, 0.1 mol) in anhydrous methanol 200mL was added dimethyl oxalate (48 g, 0.4 mol) at room temperature in a period of 30 min with stirring. Sodium methoxide (12 g, 0.22 mol) was added in a rate such that the methanol continues to reflux. The reaction mixture was stirred for 4 h and the course of the reaction was monitored with TLC. After the disappearance of the starting material, the reaction mixture was cooled to 5–10 °C. The pH value of the reaction mixture was adjusted to 5–6 with aqueous hydrochloric acid (2 N) and the mixture was maintained at this temperature with a water bath. The solvent was removed in vasuo, and the residue was dissolved in ethyl acetate (1500 mL). The solution was cooled to 10–15 °C, and the pH value was adjusted to 3 with hydrochloric acid (2 N). Water (200 mL) was added, the mixture was stirred, and organic layer was separated. The organic layer was washed with aqueous saturated sodium carbonate to adjust the pH value of organic phase to 8. Finally, 100 mL of water was added, and the organic phases was stirred for 30 min. The organic layer was then separated, and was concentrated to give compound 4 (26.7 g, 81%).

#### Synthesis of 1-(2,2-dimethoxyethyl)-5-methoxy-6-(methoxycarbonyl)-4-oxo-1,4-dihydropyridine-3-carboxylic acid (5)

A solution of compound 4 (32 g, 0.1 mol) in methanol (150 mL) was added to flask under argon atmosphere. The mixture was cooled to 0 °C with ice-water bath. To this mixture, was added anhydrous LiOH (7.2 g, 0.3 mol) in portions. The solution became turbid during the addition of process of addition of LiOH. The internal temperature of the reaction system was controlled between 0 and 5 °C with ice-water bath. TLC analysis showed the completion of the reaction after 1 h. The pH value was adjusted to 6–7 with slow addition of hydrochloric acid (2 N) at 0–5 °C. Methanol was removed in vacuo. The residue was dissolved with ethyl acetate (200 mL), and the pH value was adjusted to 1–2 via slow addition of hydrochloric acid (2 N). The temperature was maintained at 0 °C during the addition. Organic layer was separated after for 20 min, and the water phase was extracted with ethyl acetate (50 mL × 3). The combined organic layer was washed once with brine (20 mL), and was concentrated to give crude product. Recrystallization of the crude product with methanol gave compound 5 (17.6 g, 59%). ^1^H NMR (400 MHz, CDCl_3_): δ 8.42 (s, 1H), 4.54 (t, *J*^1^ = 4.0 Hz, *J*^2^ = 4.0 Hz, 1H), 4.14 (d, *J* = 8.0 Hz, 2H), 4.03 (s, 6 H), 3.42 (s, 6 H). ^13^C NMR (100 MHz, CDCl3): δ 174.82, 165.83, 161.58, 148.69, 145.25, 136.35, 116.63, 102.27, 60.93, 57.29, 55.89, 53.70.

#### Synthesis of (4R,12aS)-7-methoxy-4-methyl-6,8-dioxo-3,4,6,8,12,12a-hexahydro-2 H-pyrido[1’,2’:4,5]pyrazino[2,1-b][1, 3]oxazine-9-carboxylic acid (7)

To a reaction flask were added compound 5 (310 g, 1.0 mol) and anhydrous formic acid (1000 mL). The reaction was carried out at 65 °C with stirring under argon atmosphere. The course of the reaction was monitored with TLC. After the completion of the reaction in about 3 h, formic acid was removed in vacuo, and the temperature was kept below 45 °C. The resulting crude oil was re-dissolved in ethyl acetate (300mL), and evaporated again to remove the remaining formic acid. The processes was repeated to ensure that formic acid was removed to the maximum extent. Finally, compound 6 was obtained with an oil. This oil was used without further purification.

To a solution of compound 6 in acetonitrile (1000 mL) was added (*R*)-3-aminobutanol (125 g, 1.4 mol), and the mixture was stirred for 10 min. The mixture was then heated to reflux for 2.5 h. The course of the reaction was monitored with TLC. After the completion of the reaction, the solvent was removed in vacuo at 40–45 °C. The residue was re-dissolved in dichloromethane (2000 mL). Water (1000mL) was added, and the pH value was adjusted to 1–2 with hydrochloric acid (2 N). The mixture was stirred for 10 min, and the organic layer was separated. The aqueous phase was extracted with dichloromethane (400 mL × 4). The combined organic phase was washed with brine (200 mL × 5), concentrated in vacuo to give crude **7**. Recrystallization with methanol gave **7** (225 g,73%).^1^H NMR (400 MHz, CDCl_3_): δ 8.43 (s, 1H), 5.30 (t, *J*^1^ = 4.0 Hz, *J*^2^ = 4.0 Hz, 1H), 5.02 (t, *J*^1^ = 4.0 Hz, *J*^2^ = 8.0 Hz, 1H), 4.41 (dd, *J*^1^ = 4.0 Hz, *J*^2^ = 4.0 Hz, 1H), 4.27 (dd, *J*^1^ = 8.0 Hz, *J*^2^ = 4.0 Hz, 1H), 4.08 (s, 3 H), 4.03–3.99 (m, 2H), 2.25–2.16 (m, 1H), 1.56 (d, *J* = 12.0 Hz, 1H), 1.39 (d, *J* = 8.0 Hz, 3 H).^13^C NMR (100 MHz, CDCl_3_): δ 176.39, 165.85, 155.00, 153.90, 142.78, 130.66, 116.08, 75.97, 62.65, 61.48, 53.89, 44.93, 29.37, 16.06.

#### Preparation of compound 8

In a 500mL reaction flask, 7 (6 g, 0.02 mol), 3-aminophenylacetylene (3.51 g, 0.03 mol), HATU (11.4 g, 0.03 mol), DIPEA (7.8 g, 0.06 mol), and DMF (250mL) were combined at room temperature. The mixture was stirred for 24 h under a nitrogen atmosphere, and the reaction progress was monitored using TLC. Upon completion, confirmed by a light brown color, the reaction solution was poured into 200 mL of water, leading to the precipitation of a solid. The solid was separated, drained, and dried to yield Compound 8 with a 76% yield (6.1 g).^1^H NMR (400 MHz, DMSO-*d*_*6*_) δ 12.55 (s, 1H), 8.70 (s, 1H), 7.96 (s, 1H), 7.60 (d, *J* = 8.0 Hz, 1H), 7.38 (t, *J*^*1*^ = 8.0 Hz, *J*^*2*^ = 8.0 Hz, 1H), 7.22 (d, *J* = 8.0 Hz, 1H), 5.39 (dd, *J*^*1*^ = 4.0 Hz, *J*^*2*^ = 4.0 Hz, 1H), 4.80–4.70 (m, 1H), 4.62 (dd, *J*^*1*^ = 4.0 Hz, *J*^*2*^ = 4.0 Hz, 1H), 4.40 (dd, *J*^*1*^ = 8.0 Hz, *J*^*2*^ = 4.0 Hz, 1H), 4.21 (s, 1H), 3.98 (d, *J* = 4.0 Hz, 1H), 3.90–3.86 (m, 1H), 3.84 (s, 3 H), 2.09–1.93 (m, 1H), 1.53 (d, *J* = 12.0 Hz, 1H), 1.29 (d, *J* = 8.0 Hz, 3 H). ^13^C NMR (100 MHz, DMSO-*d*_*6*_): 173.72, 161.81, 155.11, 152.74, 143.11, 138.52, 132.26, 130.35, 129.50, 127.03, 122.46, 122.31, 120.31, 118.59, 117.42, 83.24, 80.78, 75.78, 61.71, 60.21, 52.34, 29.16, 15.67.

#### General procedure for the preparation of 9 and 10

In a flask, Compound 8 (3 mmol), substituted azide (3.6 mmol), *t*-butanol (75 mL), water (75 mL), tetrahydrofuran (75 mL), copper sulfate (1.2 g, 6 mmol), and sodium ascorbate (0.36 g, 1 mmol) were sequentially added. The mixture was heated to reflux for 10 h. After completion, the reaction mixture was extracted with dichloromethane (150 mL x 2), and the combined organic layer was washed with brine (50 mL x 4), dried over magnesium sulfate, and concentrated in vacuo to yield the crude product. Recrystallization in ethyl acetate produced the pure compound suitable for further characterization and anti-tumor studies.

The spectroscopic characterization of compounds 9a-9z and 10a-10n is provided as Supporting Information Data.

### Biological study

#### Cell viability assay

CCK8 assay was used to measure cell viability. Cells with a density of 1 × 10^4^ cells/well were seeded on the 96-well plates. After adhesion, cells were treated with different diluted compounds or vehicle control DMSO and continue cultured for 48 h. Then, CCK8 reagent was added for one hour incubation at 37 °C with 5% of CO_2_. Absorbance was measured using a microplate spectrophotometer (Thermo) at 450 nm. The ratio of cell viability of control was taken as 100%. For IC_50_, cells were treated with different concentrations of compounds (0, 0.5 µM, 2 µM, 8 µM, 16 µM, 32 µM) for 48 h and cell viability was determined to calculate the inhibition percentage. Then IC_50_ of the compounds were investigated using the prism statistical software.

#### Live and dead cells measurement

PC-9 and H460 cells with a density of 5 × 10^3^ cells/well were seeded on the 96-well plates. Then different concentrations (0, 5 µM, 10 µM, 20 µM) of 9e or 9p were treated for 24 h. Cells were then stained with the LIVE/DEAD Assay Kit, observed and photographed using the fluorescent microscope.

#### Plate clone formation assay

PC-9 and H460 cells were seeded into 6-well plates at a density of 100–500 cells/well. After 10 days culture, cells were added with 9e or 9p at different concentrations (0, 2 µM, 4 µM, 8 µM, 16 µM, 32 µM) for 48 h. Then cells were fixed by 4% paraformaldehyde and stained by Giemsa dye. An optical microscope was used to photograph cells and count the clone numbers.

#### Apoptosis assay

PC-9 and H460 cells were cultured in 6-well plates with a density of 3 × 10^5^ cells/well. Different concentrations of **9e** or **9p** were added to cells for 48 h respectively. The concentrations were 0, 2, 4, and 8 µM for PC-9 cells and 0, 4, 8 and 16 µM for H460 cells. After treatment, Annexin V-FITC Apoptosis Detection Kit was used to determine the apoptotic ratio and FlowJo software v10 was used to analyze of the results.

#### Cell cycle assay

PC-9 and H460 cells were cultured in 6-well plates with a density of 3 × 10^5^ cells/well. Cells were treated with different concentrations (0, 4, and 8 µM for PC-9 cells and H460 cells) of **9e** or **9p** for 48 h respectively. Then the cell cycle was determined by PI staining with the flow cytometer.

#### Cellular ROS measurement

PC-9 and H460 cells were cultured in 96-well plates in a density of 5 × 10^3^ cells/well. Different concentrations (0, 5 and 10 µM) of **9e** or **9p** were added to cells respectively for 24 h. After treatment, cells were stimulated with 10 µM DCFH-DA for 30 min at 37 °C, then observed and photographed using a fluorescent microscope.

#### Western blot

Protein expression levels were measured by western blot. PC-9 cells were cultured in 12-well plates and different concentrations (0, 2, 4, 8µM) of **9e** and **9p** were added for 48 h. Proteins were extracted from whole cells using radioimmunoprecipitation assay (RIPA). Buffer containing protease/phosphatase inhibitor cocktail (CST). Sodium dodecyl sulfate (10-15%) polyacrylamide gel electrophoresis and nitrocellulose membranes (Millipore) were used to separate and collect proteins. Antibodies used include, LC3 (3868s, CST), Caspase3 (9662, CST), cyclin D (2922s, CST), cyclin E (20808s, CST), γ-H2AX (9718s, CST), and β-actin (4967s, CST).

#### Statistical analyses

Data were conducted using Graph pad Prim. A two-tailed Student’s t-test or one-way analysis of variance followed by a Student-Newman-Keuls (SNK) test to assess significant differences. Values of *P* < 0.05 were considered statistically significant.

## Data Availability

The data sets used and analyzed during the current study are available from the corresponding author on reasonable request. We have presented all data in the form of Tables and Figure. Dolutegravir-1,2,3-triazole derivatives were synthesized in-house. Dulbecco’s modified Eagle medium (DMEM), RPMI 1640 Medium, Fetal bovine serum (FBS) and penicillin/streptomycin were purchased from Gibco (Grand Island, NY, USA).Enhanced Cell Counting Kit-8, Calcein/PI Live/Dead Viability Assay Kit, Giemsadye and Reactive Oxygen Species (ROS) Assay Kit were obtained from Beyotime Biotechnology (Shanghai, China). Annexin V-FITC/ Propidium iodide (PI) staining kit and Matrigel Matrix were provided by BD Biosciences (Franklin Lake, New Jersey, USA).
